# Study of out‐of‐field dose in photon radiotherapy: A commercial treatment planning system versus measurements and Monte Carlo simulations

**DOI:** 10.1002/mp.14356

**Published:** 2020-07-16

**Authors:** B. Sánchez‐Nieto, K. N. Medina‐Ascanio, J. L. Rodríguez‐Mongua, E. Doerner, I. Espinoza

**Affiliations:** ^1^ Instituto de Física Pontificia Universidad Católica de Chile Santiago Chile

**Keywords:** out‐of‐field dose, peripheral dose, second cancer, treatment planning systems

## Abstract

**Purpose:**

An accurate assessment of out‐of‐field dose is necessary to estimate the risk of second cancer after radiotherapy and the damage to the organs at risk surrounding the planning target volume. Although treatment planning systems (TPSs) calculate dose distributions outside the treatment field, little is known about the accuracy of these calculations. The aim of this work is to thoroughly compare the out‐of‐field dose distributions given by two algorithms implemented in the Monaco TPS, with measurements and full Monte Carlo simulations.

**Methods:**

Out‐of‐field dose distributions predicted by the collapsed cone convolution (CCC) and Monte Carlo (MC_Monaco_) algorithms, built into the commercially available Monaco version 5.11 TPS, are compared with measurements carried out on an Elekta Axesse linear accelerator. For the measurements, ion chambers, thermoluminescent dosimeters, and EBT3 film are used. The BEAMnrc code, built on the EGSnrc system, is used to create a model of the Elekta Axesse with the Agility collimation system, and the space phase file generated is scored by DOSXYZnrc to generate the dose distributions (MC_EGSnrc_). Three different irradiation scenarios are considered: (a) a 10 × 10 cm^2^ field, (b) an IMRT prostate plan, and (c) a three‐field lung plan. Monaco's calculations, experimental measurements, and Monte Carlo simulations are carried out in water and/or in an ICRP110 phantom.

**Results:**

For the 10 × 10 cm^2^ field case, CCC underestimated the dose, compared to ion chamber measurements, by 13% (differences relative to the algorithm) on average between the 5% and the ≈2% isodoses. MC_Monaco_ underestimated the dose only from approximately the 2% isodose for this case. Qualitatively similar results were observed for the studied IMRT case when compared to film dosimetry. For the three‐field lung plan, dose underestimations of up to ≈90% for MC_Monaco_ and ≈60% for CCC, relative to MC_EGSnrc_ simulations, were observed in mean dose to organs located beyond the 2% isodose.

**Conclusions:**

This work shows that Monaco underestimates out‐of‐field doses in almost all the cases considered. Thus, it does not describe dose distribution beyond the border of the field accurately. This is in agreement with previously published works reporting similar results for other TPSs. Analytical models for out‐of‐field dose assessment, MC simulations or experimental measurements may be an adequate alternative for this purpose.

## 1 INTRODUCTION

In recent decades, radiotherapy (RT) has undergone considerable development that has had a positive impact on long‐term outcomes for cancer patients. This has increased the interest in assessing the risk of radiogenic second cancers, whose induction is associated with the inevitable exposure to radiation of tissues outside the treatment field.^1^ In order to optimize the outcome, treatment regimes must be improved to not only maximize the probability of local tumor control but also to minimize the probability of radio‐induced effects to normal tissues (including the risk of cancer induction). For that, the concept of uncomplicated and cancer‐free control probability (UCFCP) has been recently proposed.^2^ This approach requires further knowledge about the carcinogenic potential and deterministic effects of the out‐of‐field radiation doses, which in turn has to be accurately assessed. Unfortunately, this latter is a complex task,^3^ and there is no established and reliable method to be currently used in the clinical routine, which poses unique challenges to clinical medical physicists.^4^

Today, the methodology used to determine dose from external beam RT includes measurements, treatment planning systems (TPSs), and Monte Carlo simulations.^5^ These methods are generally well established for therapeutic (in‐field) dose assessments but present several challenges for out‐of‐field regions. In particular, TPSs are not commissioned for out‐of‐field dose calculations, and significant uncertainties are reported outside the treatment field borders. For example, Howell et al.^6^ described that a TPS (Eclipse’s analytic anisotropic algorithm, AAA, V8.6) underestimated the dose up to 55% at 11.25 cm from the treatment field of a clinically relevant plan, compared to measurements with thermoluminescent dosimeters (TLDs). A similar result was obtained by Huang et al.,^7^ who also compared TPS (Pinnacle V9.0) dose calculations with TLD measurements. In this work, they reported a TPS underestimation of up to 100% and an overestimation of up to 14% of mean doses at peripheral organs for different IMRT plans. In another work, Joosten et al.^8^ reported differences in out‐of‐field organ mean doses of up to 100% when comparing MC simulations and TPS calculations using a superposition algorithm of Elekta’s CMS XiO V4.6. More recently, Bahreyni Toossi et al.^9^ showed similar results when comparing out‐of‐field doses calculated by the TiGRT TPS with TLD measurements.

The aim of this work was to thoroughly compare out‐of‐field photon doses predicted by two different algorithms available in the commercial Monaco TPS, with different dosimetry systems and Monte Carlo (MC) simulations. All cases studied in this work looked at 6 MV photon irradiations so that the dose associated with protons and neutrons (present in other currently available radiotherapy techniques and/or higher energies) did not have to be considered.

## 2 MATERIALS AND METHODS

Two algorithms from the commercially available Monaco (V5.11.0) were evaluated: Collapsed Cone Convolution (CCC) and Monte Carlo (MC_Monaco_). Out‐of‐field dose distributions (i.e., outside the 50% isodose) generated by Monaco were compared with measurements in the Elekta Axesse (Elekta, Stockholm, Sweden) linear accelerator (linac). Additionally, a full MC model (EGSnrc) for the Axesse linac was created, and the corresponding dose distributions in water and a voxelized phantom were obtained. The supplementary material also contains the results for the Eclipse's (V8.6) Pencil Beam Convolution algorithm (comparison with experimental measurements and a full MC model of the Varian 21EX linac‐Varian Medical Systems, Palo Alto, California).

An important part of this work (Sections 2.A.1 and 2.A.2) is focused on the assessment of the out‐of‐field doses within the region comprised between the border of the field (i.e., the 50% isodose) and ≈7 cm far from it (corresponding to an isodose of a few percentages of the dose on the beam axis region). This choice is motivated by three main reasons: (a) most TPSs are typically commissioned using out‐of‐axis profiles covering up to these distances, (b) no CT information is usually available far beyond those limits (e.g., for a prostate plan, no more than 7 cm above and below the PTV is included in the planning CT), which poses a natural restriction to the calculations performed by TPSs, and (c) approximately 75% of all out‐of‐field second cancers lay within this region.^1^ Moreover, the response‐to‐dose curves (deterministic and stochastic effects) for the healthy organs within this (medium to low dose) region is conditioned by a proper description of the dose–volume histograms (DVHs) provided by the TPS.

Having said that, TPSs are also able to produce dose distributions farther from the first 6–7 cm from the border of the field. Thus, to extend the region for the assessment, a three‐field lung plan on a voxelized phantom was considered. For that case, only the full MC model was used as the benchmark data (Section 2.A.3).

The coordinate system was selected to be consistent with the IEC convention.^10^ In this work, the horizontal coordinate axes X and Y were named as cross‐plane and in‐plane, respectively.

### 2.A. Beam configurations and treatment plans

#### 2.A.1. 10 × 10 cm^2^ square fields

Dose profiles for 10 × 10 cm^2^ square fields at a depth of 5 cm in water were calculated with both algorithms using a resolution of 1 mm^3^. A dose uncertainty of 1% was set for MC_Monaco_. The profiles covered up to 7 cm from the field edge, which approximately corresponds to the 1–2% isodose curve. A source‐to‐surface distance (SSD) setup was used. The profiles were compared with the ones measured with an ion chamber (IC) (Section 2.B.1), TLDs (Section 2.B.2), and EBT3 film (Section 2.B.3), and with the ones calculated using full MC simulations (Section 2.C). Local differences between experimental data and the TPS's calculation, relative to each particular algorithm, were calculated so that negative values imply a TPS underestimation of dose.

#### 2.A.2. IMRT prostate plan

An actual seven‐field IMRT treatment prostate plan was selected for this study. Adding up all fields at 0°, two‐dimensional (2D) dose distributions were generated at a depth of 5 cm in water by the MC_Monaco_ algorithm (the CCC algorithm running in Monaco was not commissioned for IMRT plans). A voxel size of 1 mm^3^, and a 1% uncertainty was set. The 2D dose distribution was compared with measurements on the linac using an EBT3 film (Section 2.B.3) and a 2D ion chamber matrix detector (Section 2.B.4). Local differences between measurement and MC_Monaco_, relative to the algorithm, were calculated so that negative values imply that TPS underestimates the dose.

#### 2.A.3. Three‐field lung plan for the ICRP110 phantom

A three‐field equally weighted (AP, LPO, and RAO) plan (5 × 5 cm^2^ open fields) was created in Monaco for the male ICRP110 reference phantom. The isocenter was located in the right lung. The prescribed dose was 60 Gy to the isocenter in 30 fractions. Dose calculations within the ICRP110 phantom were performed using a resolution of 2.1 mm^3^. An uncertainty of 1% was set for MC_Monaco_. Dose–volume histograms (DVHs) for 11 radiosensitive peripheral organs were calculated. Dose–volume histograms were then used for calculating maximum, minimum, and average dose to organs (maximum and minimum doses obtained by both algorithms for each organ are reported in Tables S1 and S2). The location of the center of mass (CoM) for each organ was obtained from the ICRP 110 publication.^11^ Then, the distance between the 50%‐isodose and the CoM (d_50%‐CoM_) for each organ (over the line joining the CoM and the isocenter) was calculated. The TPS´s dose distributions were compared with a full MC simulation (Section 2.C). Then, local differences, relative to the MC_EGSnrc_, were calculated so that negative values imply a TPS dose underestimation.

### 2.B. Experimental dosimetry

#### 2.B.1. Semiflex Ionization Chamber

A 0.125 cm^3^ Semiflex Ionization Chamber (PTW; Freiburg, Germany) (IC) was used to measure dose lateral profiles (Section 2.A.1) in a MP3 water phantom (PTW; Freiburg, Germany). Percentage Depth Dose (PDD) curves for the validation of the MC model (Section 2.C) were also measured for the same 10 × 10 cm^2^ field. PDD and lateral profiles were normalized to the maximum and central axis dose, respectively.

#### 2.B.2. Thermoluminescent dosimetry

LiF:Mg,Ti (TLD‐100) (ThermoEberline LLC, Oakwood Village, Ohio) chips of 3.2 × 3.2 × 0.89 mm^3^ were used (see Annex S1). TLD‐100 dose calibration factor was obtained for a 6 MV nominal energy photon beam. As relevant spectral variations exist outside the field, and these variations affect TLD dose response, the energy dependency correction model devised by Kron et al.^12^ and parameterized by Duggan et al.^13^ was applied. Mean energy values at each TLD position were calculated by MC simulations (see Section 2.C for more details).

TLDs were used to generate dose profiles for the 10 × 10 cm^2^ square fields (Section 2.A.1). The chips were placed forming three parallel lines with a distance between their centers of 1 cm. The readings corresponding to the three parallel crystals (at the same distance to the beam axis) were averaged. The described TLD array, sandwiched by 1 cm of a water equivalent bolus material, was irradiated inside a solid water phantom (RW3 PTW; Freiburg, Germany) at 5 cm depth with 10 cm of total backscattering material.

#### 2.B.3. Film dosimetry

GAFChromic EBT3 films (all from the same batch) were used to measure dose profiles (with film strips of size 4.0 × 25.4 cm^2^) as described in Section 2.1.1 (however, only dose values up to 7 cm from the border of the film were considered as measurements got very noisy beyond that distance). The films were irradiated under conditions similar to the ones employed for the TLDs: within the RW3 solid water phantom at 5 cm depth with 10 cm of total backscattering material. A high number of monitor units (900 MU) was used for the generation of dose profiles in order to ensure out‐of‐field dose values above 1 cGy (the threshold sensitivity of EBT3 films). For the verification of the IMRT treatment, a film of 20.3 × 25.4 cm^2^ was used.

The digitalization was done with the *EPSON® 11000XL Pro* scanner (Epson, Suwa, Nagano, Japan) in transmission mode. The orientation of the film was the same for all measurements and scanning procedures (landscape). The film calibration was done with a 6 MV photon beam, a 0.6 cm^3^ Farmer chamber (PTW, Freiburg, Germany), and a combined protocol based on the works by Devic et al.^14^ (from 400 cGy) and Tamponi et al.^15^ (up to 400 cGy). The latter minimizes the energy dependence for the low dose region.

#### 2.B.4. 2D ion chamber matrix detector

A 2D ion chamber matrix detector was also used to measure the dose distributions corresponding to the IMRT prostate plan described in Section 2.A.2. The Octavius Detector 1500 (PTW, Freiburg, Germany), consistent of 1405 plane–parallel vented ionization chambers of size 4.4 × 4.4 × 3 mm^3^ (0.06 cm^3^), covering an area of 27 × 27 cm^2^, was chosen. The matrix was placed under 5 cm of the RW3 phantom.

### 2.C. Monte Carlo simulations

The Monte Carlo simulation system BEAMnrc,^16^ built on the EGSnrc code system,^17^ was used to create a detailed model of the Elekta Axesse with the Agility collimation system. The geometry and composition of the accelerator head were based on technical drawings provided by the manufacturers. Such models include the target, primary collimator, flattening filter, monitor chamber, mirror, collimating jaws, and MLC.

A Gaussian spectrum of the electron beam (centered at 6.500 ± 0.212 MeV with a focal spot of diameter 0.15 cm^18^) impinging the target was simulated.

Firstly, phase space files were scored at a distance from the source of 100 cm. To not affect the dose deposited outside the treatment field, the Directional Bremsstrahlung Splitting (DBS) technique was not considered. Doses were calculated with DOSXYZnrc in a water phantom using 2 × 10^8^ histories from the phase space files (also part of the EGSnrc toolkit). In order to improve the calculation efficiency, the photon splitting technique with a split factor of 40 was used.^19^ For all simulations, the electron and photon transport cutoff energies were 0.512 and 0.001 MeV, respectively. Dose distributions for the configurations described in Section 2.A.1 were simulated in the water phantom (1.0 g/cm^3^ density) of 30.0 × 30.0 × 25.6 cm^3^ (voxel size of 0.3 × 0.3 × 0.1 cm^3^ with the shorter dimension along the beam axis Z). In order to validate the MC model, simulated PDDs and lateral profiles were compared to those measured with the Semiflex IC in the water phantom. Additionally, a mean energy profile for a 6 MV 10 × 10 cm^2^ field, at 5 cm depth in water, was calculated to correct for the TLD energy dependence.

Secondly, DOSXYZnrc was used for the dose scoring (4 × 10^8^ histories per beam) in the ICRP110 male phantom for the lung irradiation (Section 2.A.3). The voxel size was 2.137 × 2.137 × 8.0 mm^3^. From now onward, these MC simulations will be referred to as MC_EGSnrc_.

## 3 RESULTS

### 3.A. Monte Carlo validation

**Fig. 1:**
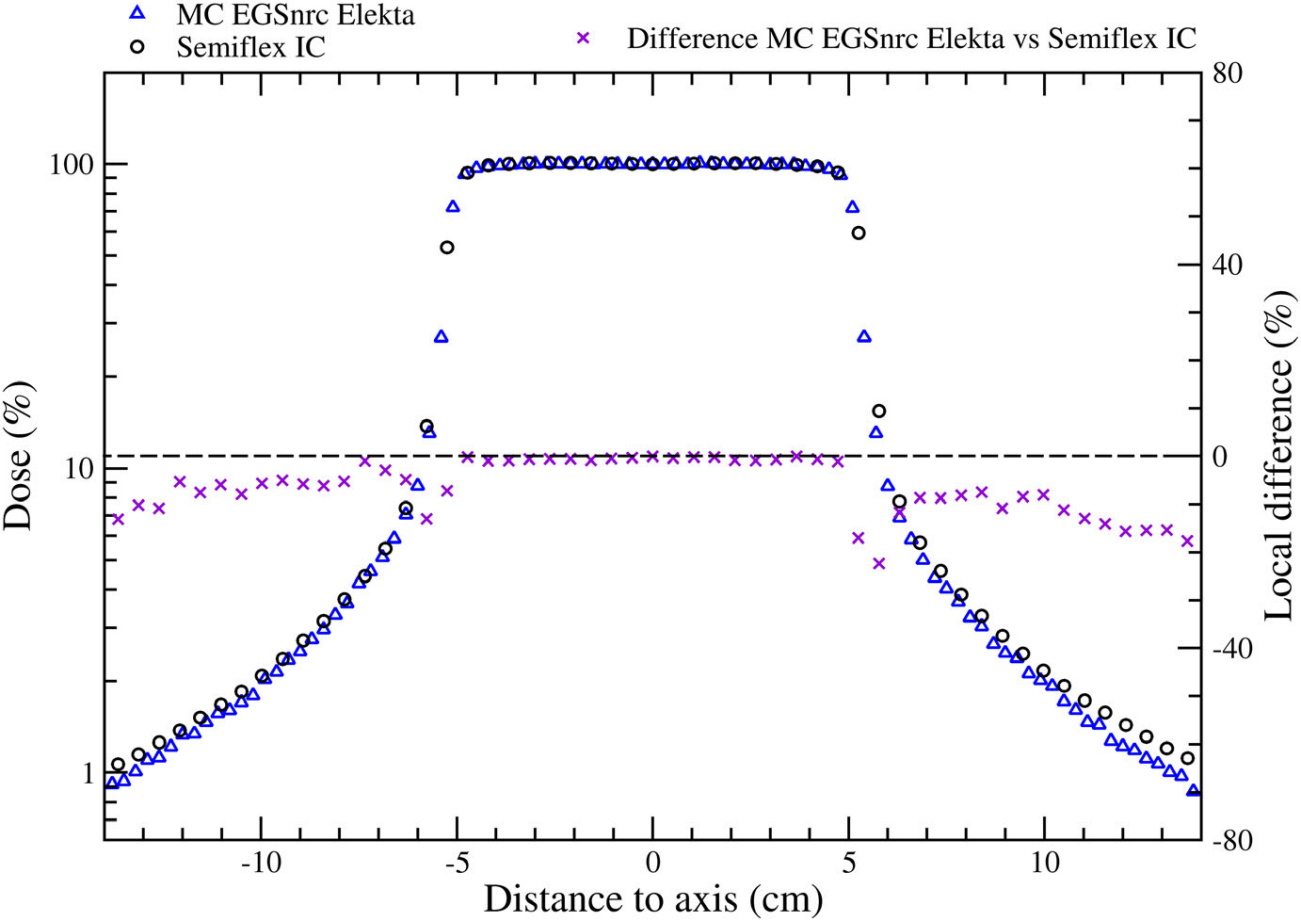
Semiflex IC and MC in‐plane lateral profiles (left vertical axis) and local differences (right vertical axis). See Fig. S1 for the in‐plane profiles in the Varian linac. [Color figure can be viewed at wileyonlinelibrary.com]

Local differences between MC_EGSnrc_ and IC measurements, relative to the latter, were calculated. For the PDDs, local differences increased with depth up to a maximum of 2% (see Fig. S0). Figure 1 depicts measured and MC_EGSnrc_ calculated in‐plane profiles as well as the local differences.

### 3.B. Simulated spectra

**Fig. 2:**
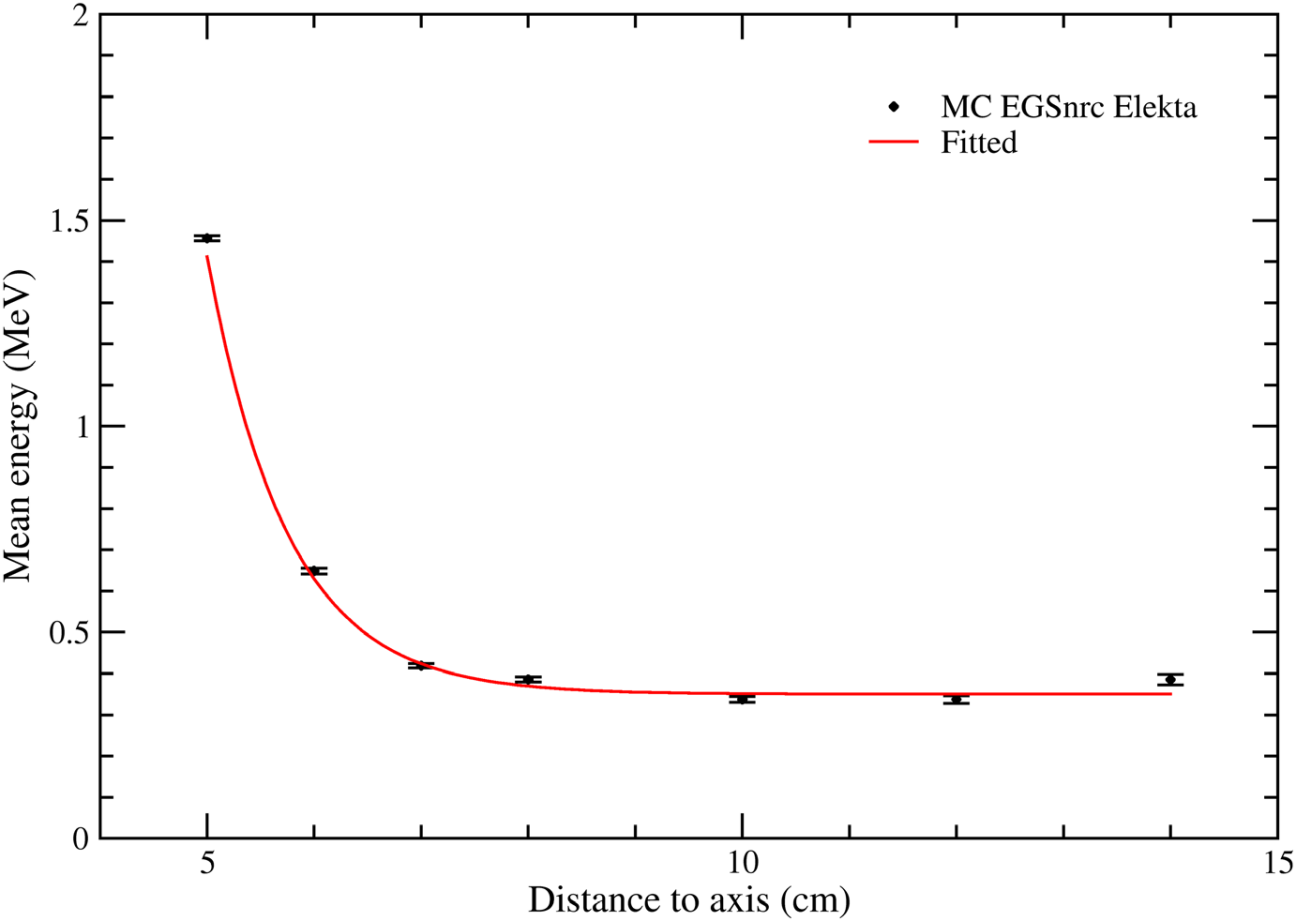
Average energy of a 6 MV photon beam of an Elekta Axesse linac at 5 cm depth in water as a function of the distance to the central axis for a 10 × 10 cm^2^ field. The increase of the energy at the farthest point has also been described in other works,^20^, ^21^ and it can be explained by the increase of head leakage and collimator scatter.^22^ An SSD = 95 cm setup was simulated. See Fig. S2 for the average energy obtained for the Varian linac. [Color figure can be viewed at wileyonlinelibrary.com]

The average photon energy ⟨E⟩ calculated from MC simulations is depicted in Fig. 2 as a function of the distance to the central axis of the beam. Solid lines correspond to the fitted interpolation model $\left\langle E\right\rangle =Ae^{\left(\frac{‐(x‐5)}{t}\right)}+E_{0}$, where A, t, and E_0_ are the fitting parameters. The parameters values were A = 1,103 ± 0,024 MeV, *t* = 0,751 ± 0,045 cm^−1^, and E_0_ = 0,354 ± 0,011 MeV. This expression was further used to calculate the mean energy at the position of the TLD chips during the profile measurements.

### 3.C. 10 × 10 cm^2^ square fields

**Fig. 3:**
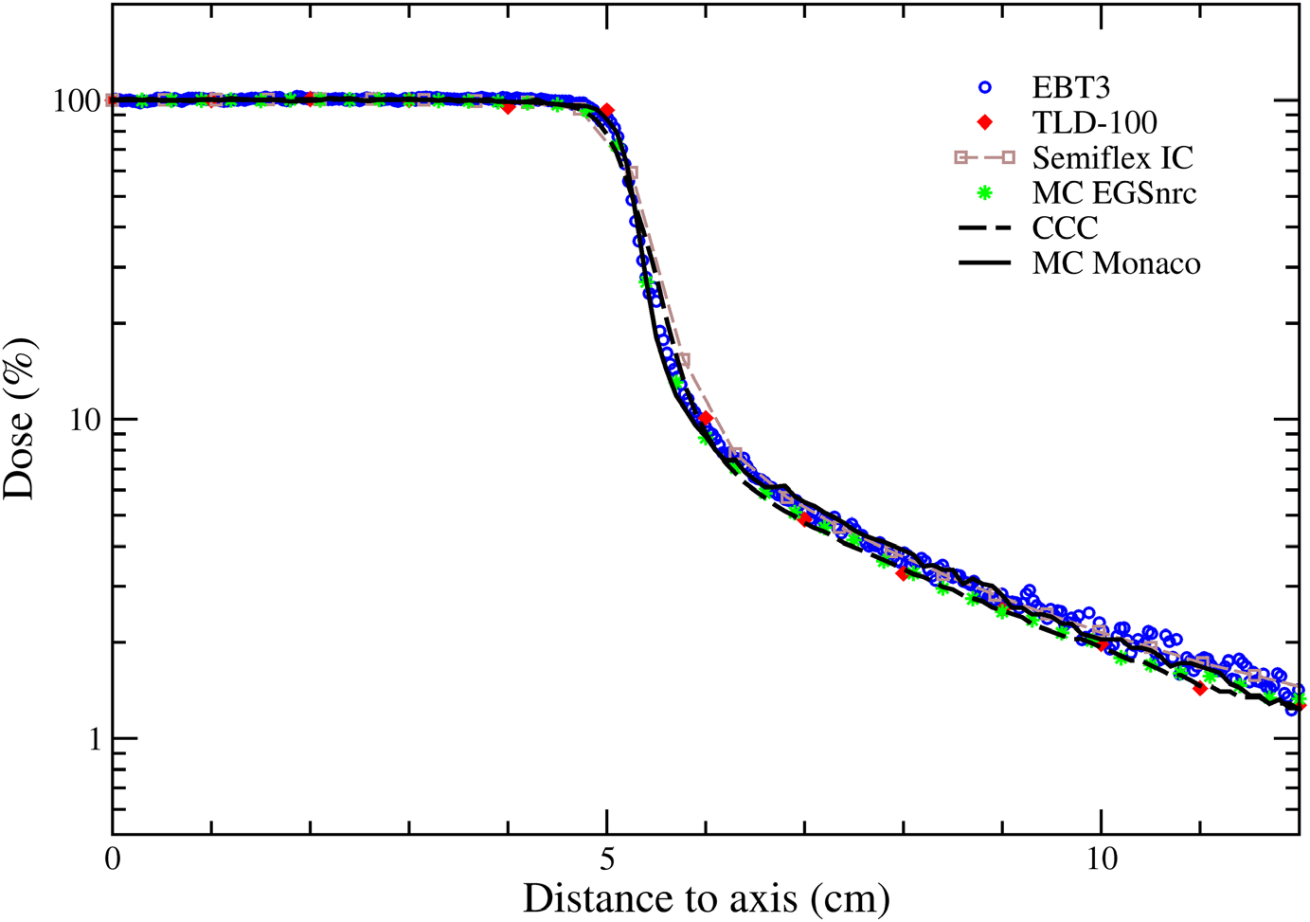
Half in‐plane dose profiles calculated with Monaco and experimental data for the Elekta linac. Profiles calculated with Eclipse's PBC algorithm and experimental data obtained for the Varian linac are shown in Fig. S3. [Color figure can be viewed at wileyonlinelibrary.com]

**Fig. 4:**
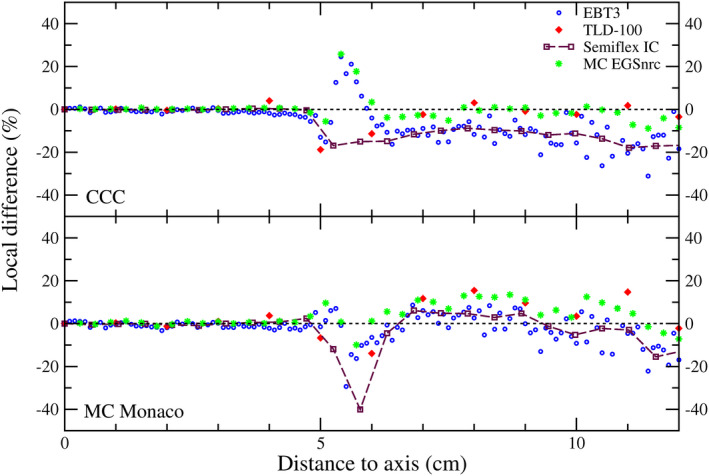
Local differences between calculated (Monaco’s CCC and MC_Monaco_) and experimental (EBT3, TLD‐100, Semiflex IC and MC_EGSnrc_) in‐plane dose profiles, relative to the algorithm. Negative values indicate that the algorithm underestimates the measured dose. The values corresponding to the Eclipse’s PBC algorithm and the experimental data obtained for the Varian linac are shown in Fig. S4. [Color figure can be viewed at wileyonlinelibrary.com]

Figure 3 shows half in‐plane dose profiles for both algorithms and the experimental data. Relative local differences are depicted in Fig. 4.

Within the region between the field edge (50% isodose) and the 20% isodose (i.e., the out‐of‐field part of the penumbra), an over‐ and underestimations with respect to EBT3 occur for both algorithms. The overestimation was of up to 25% for CCC and 7% for MC_Monaco_ and the underestimation was of up to 15% for CCC and 29% for MC_Monaco_.

Beyond the 5% isodose, CCC showed an underestimation of absorbed dose. Relative differences with the IC ranged from 9% to 18% (on average 13%). For the same region, MC_Monaco_ calculations agreed with measurements within the uncertainties (the differences fluctuate between ±5%), except for a single point at 11.6 cm from the isocenter (the largest distance shown), where an underestimation of around 15% (similar to CCC) was found.

### 3.D. IMRT prostate plan

**Fig. 5:**
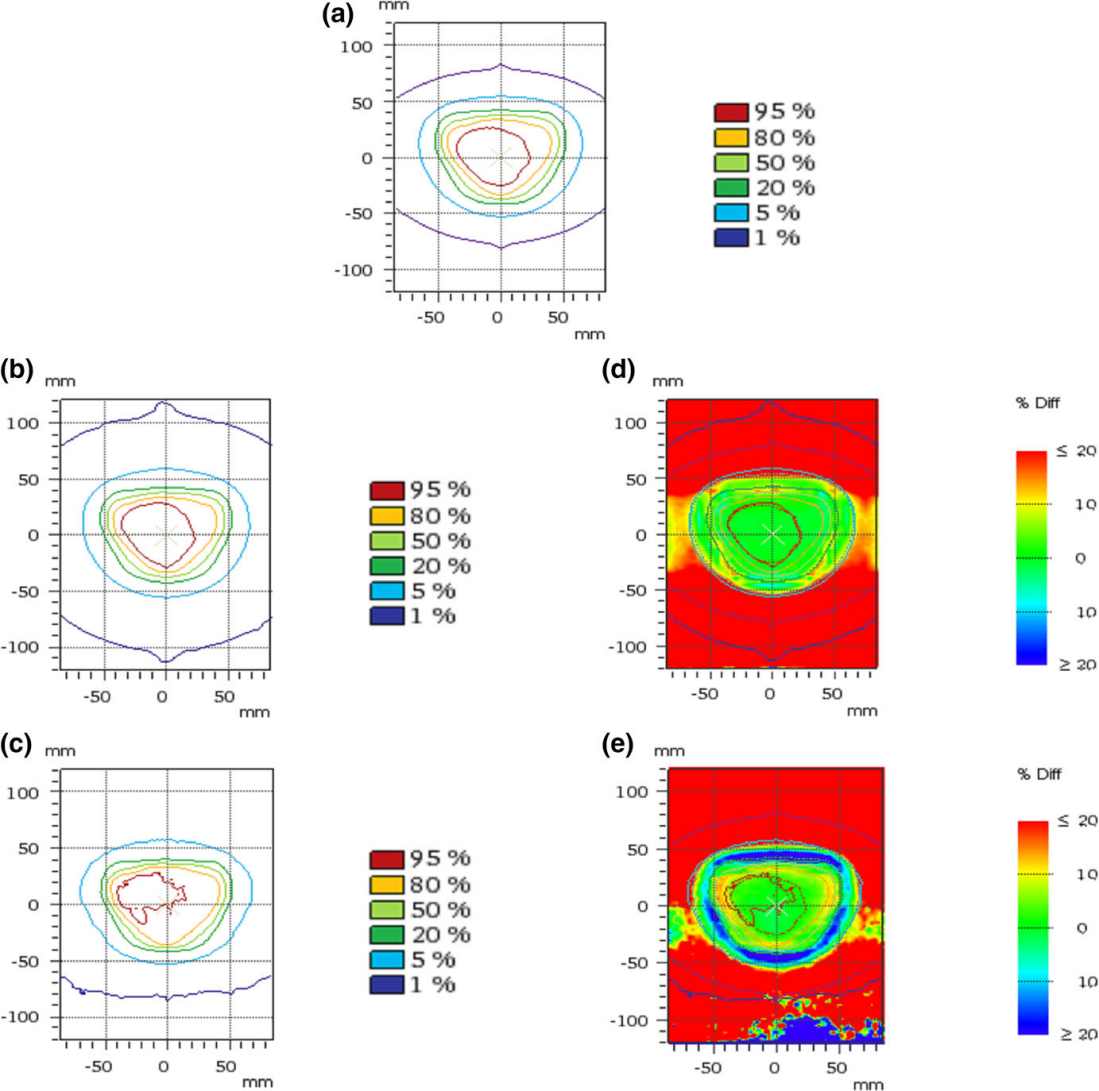
IMRT dose distribution analysis for MC_Monaco_: (a) dose distributions calculated by the TPSs; (b) dose distribution measured with the Octavius Detector 1500; (c) dose distribution measured with the EBT3 film; (d) local differences between (a) and (b), with respect to (a); (e) local differences between (a) and (c), with respect to (a). Note: the lower part of (e) presents “noisy” blue‐to‐yellow regions that are a consequence of an error in the scanning process of the film and should not be taken into account in the analysis. [Color figure can be viewed at wileyonlinelibrary.com]

Figures 5 shows the 2D dose distribution for the IMRT prostate plan calculated with the MC_Monaco_ algorithm (a), together with the ones measured with the 2D matrix detector (b) and the EBT3 film (c). The corresponding local differences, with respect to the Monaco´s calculation, are also displayed (d and e). It is worth noting that the central vertical axis in Fig. 5 corresponds to the in‐plane direction (the direction of the profiles and the local relative differences shown in Figs. 3 and 4, respectively). A very good agreement between TPS and measured dose distributions was observed within the 50% isodose and an underestimation of dose of ≥20% was observed for almost all the regions beyond the 5% isodose (light blue). This underestimation is less severe in some regions in the cross‐plane direction. Large over‐ and underestimations of dose were observed between the 50% and the 5% isodoses in the comparison with the EBT3 film (e).

### 3.E. Three‐field lung plan for the ICRP110 phantom

**Fig. 6:**
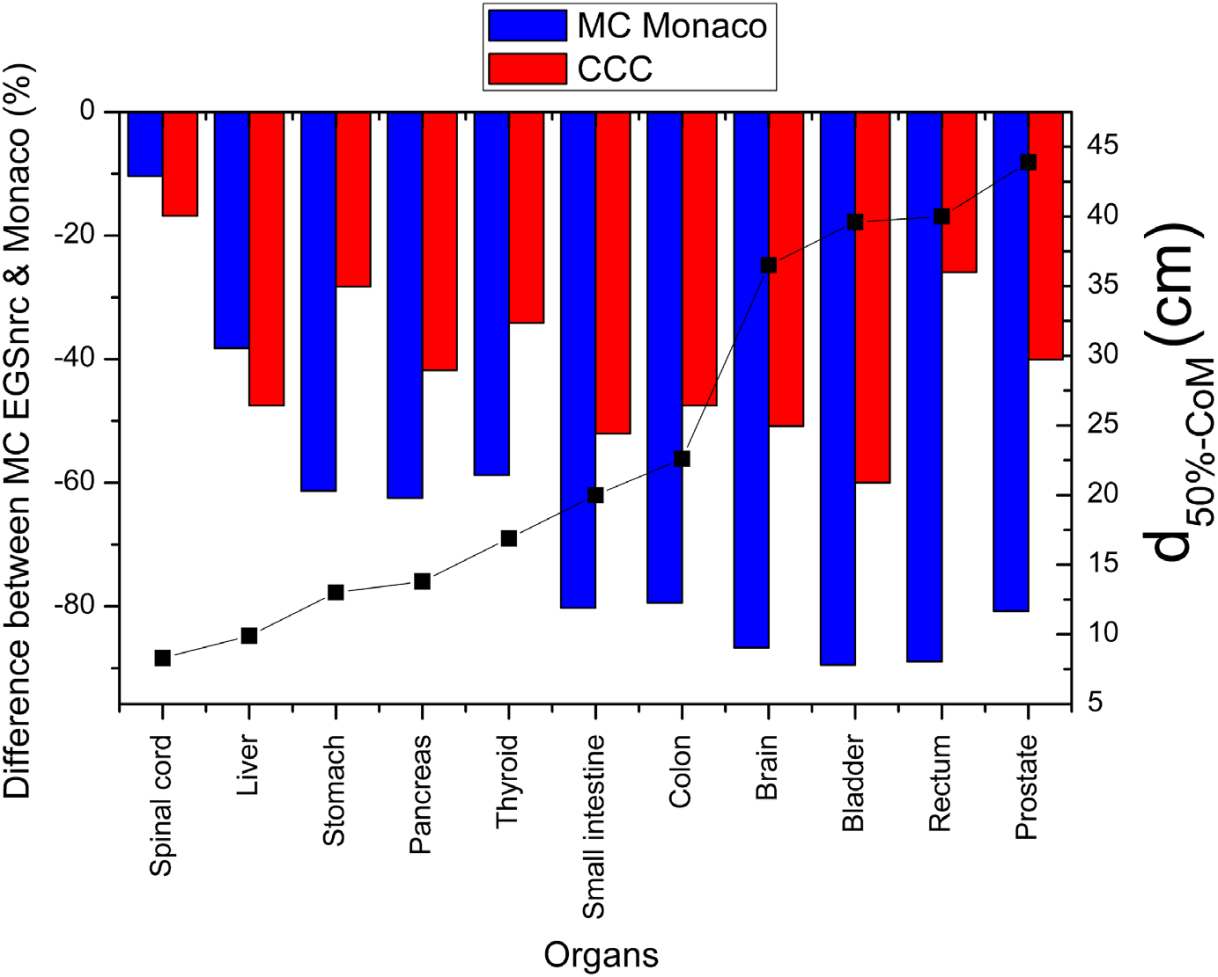
Color bars represent relative differences (left vertical axis) in mean absorbed dose between Monaco and MC_EGSnrc_ relative to the latter, for different organs. d_50%‐CoM_ represents the distance from the CoM of each organ to the field edge (right vertical axis). These distances are plotted as solid black squares. [Color figure can be viewed at wileyonlinelibrary.com]

Differences between average dose to organ for both Monaco's algorithms and MC_EGSnrc_, relative to the latter, are depicted in Fig. 6. Both algorithms underestimate the dose. The underestimation by MC_Monaco_ increases in magnitude for larger distances from the border of the field. Differences in mean dose to organ with respect to MC simulations increased up to ≈90% for MC_Monaco_ and ≈60% for CCC.

## 4 DISCUSSION

### 4.A. Algorithms' performance

The assessment of dose in the out‐of‐field part of the penumbra is critical because (a) most second cancers arise there,^1^ and (b) deterministic radiation side effects on the nearby organ at risk usually constraint the planned dose to the tumor. In this work, both algorithms failed in defining this region properly. More specifically, both under‐ and overestimations were observed when compared to the film (the system used without suffering from volume effect — see Section 4.D). An underestimation of dose leads to an underestimation of the risk of stochastic and deterministic effects. An overestimation could also be a critical issue during inverse planning optimization, as dose constraints could be imposed on the organ at risk lying close to the 50% isodose and, consequently, the optimized plan might carry some under dosage to the tumor. Conversely, the overestimation would imply a conservative assessment of both stochastic and deterministic effects.

Despite the fact that both algorithms (commissioned by accomplishing the manufacturer recommendations on length of the profiles) could reproduce the in‐field region properly, non‐negligible differences with experimental measurements (done with the same IC used for commissioning) were observed in the out‐of‐field region. This is shown in Fig. 4. This is a somewhat expected result as the parameterization of the algorithms during commissioning tries to optimize its operation within the field. However, in order to evaluate the performance of an algorithm, it is necessary to look at its ability to reproduce the experimental data of the detector used for the algorithm's commissioning. Thus, we cannot expect the algorithms to perform better than the IC. Even if the IC had been a perfect detector (i.e., without volume effect and energy dependence — see Section 4.D), the differences between algorithms and IC would have remained (the algorithms have limitations on reproducing the experimental data as compromises on the goodness of the fitting have to be balanced between the in‐ and out‐of‐field regions).

Figure 5 showed that qualitatively similar results, compared to the ones for the square field of Fig. 4, are also present in the studied IMRT case: (a) large underestimations of dose were observed beyond the 5% isodose and (b) over‐ and underestimations are observed between the 50% and the 5% isodose in comparison with the EBT3 film. As mentioned above, the latter can be explained by the good spatial resolution of the film, which does not show the volume effects shown by the array of ICs in the Octavius detector. We hypothesize that the smaller dose underestimation in the cross‐plane direction (compared to the in‐plane direction) shown in Fig. 5 (d and e) may be explained by imperfect modeling of the linac's collimation system by the TPS.

Note that results depicted in Fig. 4 are relative differences with respect to each algorithm. This was done to make differences comparable among all the benchmark dosimetric systems used for each algorithm. When the differences between the CCC algorithm and IC measurements are calculated relative to IC, results are almost the same. In particular, our reported underestimation of 13% changes to 12%. For the MC_Monaco_, the local differences at 11,6 cm from the axis changes from −15% to −13%. The latter are the results which are comparable with the local differences depicted in Fig. 6 (relative to the MC_EGSnrc_ benchmark data). The study with the voxelized phantom (Section 2.A.3 and Fig. 6) allowed us to assess the behavior of CCC and MC_Monaco_ at larger distances from the field edge than those evaluated by IC, TLDs, or film. Note that the regions assessed in Figs. 3 (or 4) and 6 are adjacents, and that results for the abutting points are consistent (i.e., underestimation of the dose by both algorithms, with a better performance of MC_Monaco_). For organs with CoM placed farther than ≈13 cm (e.g., stomach) from the border of the field, CCC performs better than MC_Monaco_.

To our knowledge, there are no other published works studying the performance of Monaco in the out‐of‐field region. As mentioned in the introduction, there are only a few works showing out‐of‐field dose comparisons of specific TPSs (Pinnacle, Eclipse, XiO, and TIGRT) with measurements (IC or TLD) or MC simulations.^6^, ^7^, ^8^, ^9^ They consider different ranges of distances and ways to calculate differences in the dose. Even though these differences make direct comparisons difficult, we may say that our relative local differences are, in general, consistent with what they observed (e.g., at 3,75 cm from the border of the field, Howell et al.^6^ measured an underestimation of 16% compared with our result of ≈10% for the CCC algorithm). In essence, the differences are of the same order of magnitude, and they increase at larger distances from the treatment field. To put these relative differences in perspective, it is worth mentioning that a relative local underestimation of 10% (with respect to the IC) at 3,5 cm (isodose of ≈2%) translates into 14 cGy for a treatment with a prescribed dose of 70 Gy. In contrast, the same 10% is overestimated by CCC (with respect to EBT3) at the border of the penumbra (20%), which translates into 1.4 Gy.

There are analytical models developed explicitly for out‐of‐field dose estimation,^23^, ^24^, ^25^, ^26^, ^27^, ^28^ some of them been implemented within a software application.^23^, ^26^ This work motivates further development of these models, which should consider the current RT techniques and must be implemented as a clinically useful tool.

### 4.B. Monte Carlo validation

As was shown in Fig. 1, there is an excellent agreement between the dose calculated with our MC model and the dose measured with the Semiflex IC in the plateau of the lateral profile. The significant discrepancies observed in the penumbra region can be explained by the volume averaging effect of the IC. Beyond the field edge, an increasing difference of up to almost 20% was observed between MCEGS_nrc_ and IC measurements. These differences are similar to those reported by Joosten et al.^29^ (without accounting for the shielding component) as well as Kry et al.^30^ and Bednarz and Xu^31^ (with more complex MC models accounting for the shielding component).

### 4.C. Simulated spectra

To our knowledge, there is no published data of the photon spectrum mean energy as a function of the distance with the same conditions as shown in Fig. 2, so a direct comparison is not possible. However, our results are very similar to other published results based on similar conditions.^20^ According to the mean energy dependence with distance to the axis, the energy corrections applied to the TLDs were of <3%, in agreement with Kry et al.^30^

### 4.D. Dosimetric systems' performance

According to TG158, ICs have many good traits for measuring out‐of‐field doses.^4^ This report mentions logistical issues, not applicable to our setup conditions. Additionally, it mentions the energy dependence, which, according to the manufacturer of our chamber, would be within ±4% (100 keV‐60Co). This uncertainty would propagate up to a maximum of ±5% in the local differences depicted in Fig. 4. Another important issue with the IC is the volume effect, relevant for dose gradient regions. Therefore, IC would be a good dosimetric system for all out‐of‐field regions except the first few centimeters from the field edge, where a relevant dose gradient is present. Interestingly, Figs. 3, 4, 5 reveal how both algorithms reproduced the volume effect of the ICs used for their commissioning. In the penumbra region of Fig. 5, differences between MC_Monaco_'s calculations and Octavius detector’s measurements — built‐in with air chambers of 0,06 cm^3^ — are smaller than between MC_Monaco_'s calculations and EBT3 film. Even the IC chambers inside Octavius, smaller than the semiflex IC used for commissioning, do not describe the dose gradient properly in the penumbra region. The same volume effect is to be found in TLD measurements due to the size of the crystals. When TLDs are properly energy‐corrected, they may also be a good option for measuring out‐of‐field dose beyond the penumbra. Thus, among the dosimetric systems considered in this work, the ideal one for defining the region close to the field edge would be the film, as it has an excellent spatial resolution. Two concerns about the film dosimetry are the energy dependence and the signal to noise ratio. The former was minimized by the calibration methodology used in this work. The noise could be minimized, averaging over space, though compromising the spatial resolution. In Fig. 3, TLDs reported larger doses than EBT3 film and IC. These discrepancies come from the slightly different irradiation conditions (as mentioned before, TLDs were sandwiched between bolus layers). When EBT3 film was irradiated under the same conditions as with the TLDs, a lower dose was measured (see Fig. S4).

Due to the previously mentioned limitations, the choice of the dosimetry system should be made depending on the out‐of‐field region to be studied.

## 5 CONCLUSIONS

The performance for out‐of‐field dose calculations of two algorithms implemented in a commercial TPS was thoroughly evaluated by comparison with experimental measurements and full MC simulations. The evaluation was done under three different scenarios, from open square fields to IMRT. In the case of a 10 × 10 cm^2^ field, TPSs were not able to reproduce the dose gradients measured by the IC (also used for commissioning). On average, CCC underestimated the dose by ≈13% between the 5% and 2% isodoses. MC_Monaco_ underestimated the dose only from approximately the 2% isodose. Beyond the 2% isodose, differences in mean dose to organ with respect to MC simulations increased up to ≈90% for MC_Monaco_ and ≈60% for CCC. In another comparison, an IMRT prostate plan was also studied. In this case, similar results were obtained, compared to the study with an open square field, confirming the deficient performance of the TPS in the out‐of‐field region. Because of the volume effect shown by the semiflex IC and 2D IC matrix detectors, we suggest the use of film dosimetry for the commissioning and checking of dose gradient regions. In general, we have shown that the algorithms studied in this work do not give reliable values of out‐of‐field dose.

## Supporting information


**Fig S0**. PDD measured with a semiflex IC and calculated with MCEGSnrc for the Varian21EX linac (upper plot) and Elekta Asses (lower plot). (x) represent the local differences relative to measurements (right axis). SSD = 100 cm and SSD = 95 cm setups for the Varian and Elekta linacs were used, respectively.

**Fig S1.** Semiflex IC and MC in‐plane lateral profiles (left vertical axis) and local differences (right vertical axis) for the Varian linac. SSD=100 cm setup was used [Correction added on September 9, 2020, after first online publication: The Fig S1.caption have been corrected.]

**Fig S2.** Average energy of a 6 MV photon beam of a Varian 21EX linac. MC calculation was carried out at 5 cm depth in a water phantom (SSD = 100 cm). Solid lines correspond to the fitted interpolation model $〈E〉=Ae^{\left(‐(x‐5)/t\right)}+E_{0},$ where A, t, and E_0_ are the fitting parameters (A = 0.895 ± 0.037 MeV, t = 0.511 ± 0.076 cm^−1^, and E_0_ = 0.348 ± 0.016 MeV).

**Fig S3.** Half in‐plane dose profiles calculated with Eclipse and experimental data for the Varian linac.

**Fig S4.** Profiles measured with EBT3 and TLDs under more similar conditions (i.e., EBT3 underneath the same bolus used for TLDs set up).

**Table S1.** Percentage difference in dose to organs with CCC.
**Table S2**. Percentage difference in dose to organs with MC_Monaco_.

**Annex S1.** TLD procedure.

## References

[1] Diallo I , Haddy N , Adjadj E , et al. Frequency distribution of second solid cancer locations in relation to the irradiated volume among 115 patients treated for childhood cancer. Int J Radiat Oncol Biol Phys. 2009;74:876–883. 19386434 10.1016/j.ijrobp.2009.01.040

[2] Sánchez‐Nieto B , Romero‐Expósito M , Terrón JA , Sánchez‐Doblado F , Brenner DJ , Newhauser WD . Uncomplicated and Cancer‐Free Control Probability (UCFCP): a new integral approach to treatment plan optimization in photon radiation therapy. Phys Medica. 2017;47:35–41. 10.1016/j.ejmp.2017.03.025 28392313

[3] Newhauser WD , Durante M . Assessing the risk of second malignancies after modern radiotherapy. Nat Rev Cancer. 2011;11:438–448. 21593785 10.1038/nrc3069 PMC4101897

[4] Kry SF , Bednarz B , Howell RM , et al. AAPM TG 158: Measurement and calculation of doses outside the treated volume from external‐beam radiation therapy. Med Phys. 2017;44:e391–e429. 28688159 10.1002/mp.12462

[5] Newhauser WD , de Gonzalez AB , Schulte R , Lee C . A review of radiotherapy‐induced late effects research after advanced technology treatments. Front Oncol. 2016;6:13. 26904500 10.3389/fonc.2016.00013 PMC4748041

[6] Howell RM , Scarboro SB , Kry SF , Yaldo DZ . Accuracy of out‐of‐field dose calculations by a commercial treatment planning system. Phys Med Biol. 2010;55:6999–7008. 21076191 10.1088/0031-9155/55/23/S03 PMC3152254

[7] Huang JY , Followill DS , Wang XA , Kry SF . Accuracy and sources of error of out‐of field dose calculations by a commercial treatment planning system for inte sity‐modulated radiation therapy treatments. J Appl Clin Med Phys. 2013;14:186–197. 10.1120/jacmp.v14i2.4139 PMC5714363 23470942

[8] Joosten A , Matzinger O , Jeanneret‐Sozzi W , Bochud F , Moeckli R . Evaluation of organ‐specific peripheral doses after 2‐dimensional, 3‐dimensional and hybrid intensity modulated radiation therapy for breast cancer based on Monte Carlo and convolution/superposition algorithms: implications for secondary cancer risk asses. Radiother Oncol. 2013;106:33–41. 23351844 10.1016/j.radonc.2012.11.012

[9] Bahreyni Toossi M , Soleymanifard S , Farhood B , Mohebbi S , Davenport D . Assessment of accuracy of out‐of‐field dose calculations by TiGRT treatment planning system in radiotherapy. J Cancer Res Ther. 2018;14:634–639. 29893331 10.4103/0973-1482.176423

[10] IEC1217: Radiotherapy equipment‐coordinates, movements and scales IEC. No Title; 1996.

[11] ICRP . Adult Reference Computational Phantoms ICRP. Ann ICRP; 2009 10.1016/j.icrp.2009.07.004

[12] Kron T , Duggan L , Smith T , et al. Dose response of various radiation detectors to synchrotron radiation. Phys Med Biol. 1998;43:3235–3259. 9832014 10.1088/0031-9155/43/11/006

[13] Duggan L , Hood C , Warren‐Forward H , Haque M , Kron T . Variations in dose response with x‐ray energy of LiF:Mg, Cu, P thermoluminescence dosimeters: Implications for clinical dosimetry. Phys Med Biol. 2004;49:3831–3845. 15470908 10.1088/0031-9155/49/17/001

[14] Devic S , Seuntjens J , Hegyi G , et al. Dosimetric properties of improved GafChromic films for seven different digitizers. Med Phys. 2004;31:2392–2401. 15487718 10.1118/1.1776691

[15] Tamponi M , Bona R , Poggiu A , Marini P . A new form of the calibration curve in radiochromic dosimetry. Properties and results. Med Phys. 2016;43:4435–4446. 27370159 10.1118/1.4954208

[16] Rogers DWO , Walters B , Kawrakow I . BEAMnrc users manual. Report PIRS‐0509(A)revL. Ottawa, Canada: NRCC; 2019.

[17] Kawrakow I , Mainegra‐Hing E , Rogers DWO , Tessier F , Walters BRB . The EGSnrc code system: Monte Carlo simulation of electron and photon transport. National Research Council of Canada Report PIRS‐701. Ottawa, Canada: NRCC; 2019.

[18] Ureba Sánchez AM . Planificación radioterápica de intensidad modulada en un modelo de simulación explícita del transporte de partículas mediante optimización por imagen médica; 2015 http://hdl.handle.net/11441/26791

[19] Walters B , Kawrakow I , Rogers DWO . DOSXYZnrc users manual. National Research Council of Canada Report PIRS‐794revB. Ottawa, Canada: NRCC; 2005.

[20] Irazola L , Terrón JA , Bedogni R , et al. Neutron measurements in radiotherapy: a method to correct neutron sensitive devices for parasitic photon response. Appl Radiat Isot. 2017;123:32–35. 28214683 10.1016/j.apradiso.2016.12.060

[21] Pascal Hauri von . Out‐of‐Field Dose in Photon Radiotherapy: Models and Measurements; 2017 10.5167/uzh-144064

[22] Ruben JD , Lancaster CM , Jones P , Smith RL . A comparison of out‐of‐field dose and its constituent components for intensity‐modulated radiation therapy versus conformal radiation therapy: implications for carcinogenesis. Int J Radiat Oncol Biol Phys. 2011;81:1458–1464. 20950947 10.1016/j.ijrobp.2010.08.008

[23] Van Der Giessen PH . Peridose, a software program to calculate the dose outside the primary beam in radiation therapy. Radiother Oncol. 2001;58:209–213. 11166873 10.1016/s0167-8140(00)00326-1

[24] Stovall M , Weathers R , Kasper C , et al. Dose reconstruction for therapeutic and diagnostic radiation exposures: use in epidemiological studies linked references are available on JSTOR for this article: dose reconstruction for therapeutic and diagnostic radiation exposures: use in epidemiolog. Radiat Res. 2020;166:141–157. 10.1667/RR3525.1 16808603

[25] Taddei PJ , Jalbout W , Howell RM , et al. Analytical model for out‐of‐field dose in photon craniospinal irradiation. Phys Med Biol. 2013;58:7463–7479. 24099782 10.1088/0031-9155/58/21/7463 PMC4395760

[26] Sánchez‐Nieto B , Elfar R , Irazola L , et al. Analytical model for photon peripheral dose estimation in radiotherapy treatments. Biomed Phys Eng Express. 2015;1:045205.

[27] Jagetic LJ , Newhauser WD . A simple and fast physics‐based analytical method to calculate therapeutic and stray doses from external beam, megavoltage x‐ray therapy. Phys Med Biol. 2015;60:4753–4775. 26040833 10.1088/0031-9155/60/12/4753 PMC4497528

[28] Hauri P , Hälg RA , Besserer J , Schneider U . A general model for stray dose calculation of static and intensity‐modulated photon radiation. Med Phys. 2016;43:1955–1968. 27036591 10.1118/1.4944421

[29] Joosten A , Bochud F , Baechler S , Levi F , Mirimanoff RO , Moeckli R . Variability of a peripheral dose among various linac geometries for second cancer risk assessment. Phys Med Biol. 2011;56:5131–5151. 21775792 10.1088/0031-9155/56/16/004

[30] Kry SF , Titt U , Pönisch F , et al. A Monte Carlo model for calculating out‐of‐field dose from a Varian 6 MV beam. Med Phys. 2006;33:4405–4413. 17153419 10.1118/1.2360013

[31] Bednarz B , Monte XuXG . Carlo modeling of a 6 and 18 MV Varian Clinac medical accelerator for in‐field and out‐of‐field dose calculations: development and validation. Phys Med Biol. 2009;54:N43–N57. 19141879 10.1088/0031-9155/54/4/N01 PMC3376900

