# Supplementary material for: Study of out‐of‐field dose in photon radiotherapy: A commercial treatment planning system versus measurements and Monte Carlo simulations
Source: Med Phys. 2020 Jul 16;47(9):4616–25. doi: 10.1002/mp.14356 (PMC7586840; doi:10.1002/mp.14356)
Supplement: Supplementary file 1 — Fig S0. PDD measured with a semiflex IC and calculated with MCEGSnrc for the Varian21EX linac (upper plot) and Elekta Asses (lower plot). (x) represent the local differences relative to measurements (right axis). SSD = 100 cm and SSD = 95 cm setups for the Varian and Elekta linacs were used, respectively. [file MP-47-4616-s001.pdf]

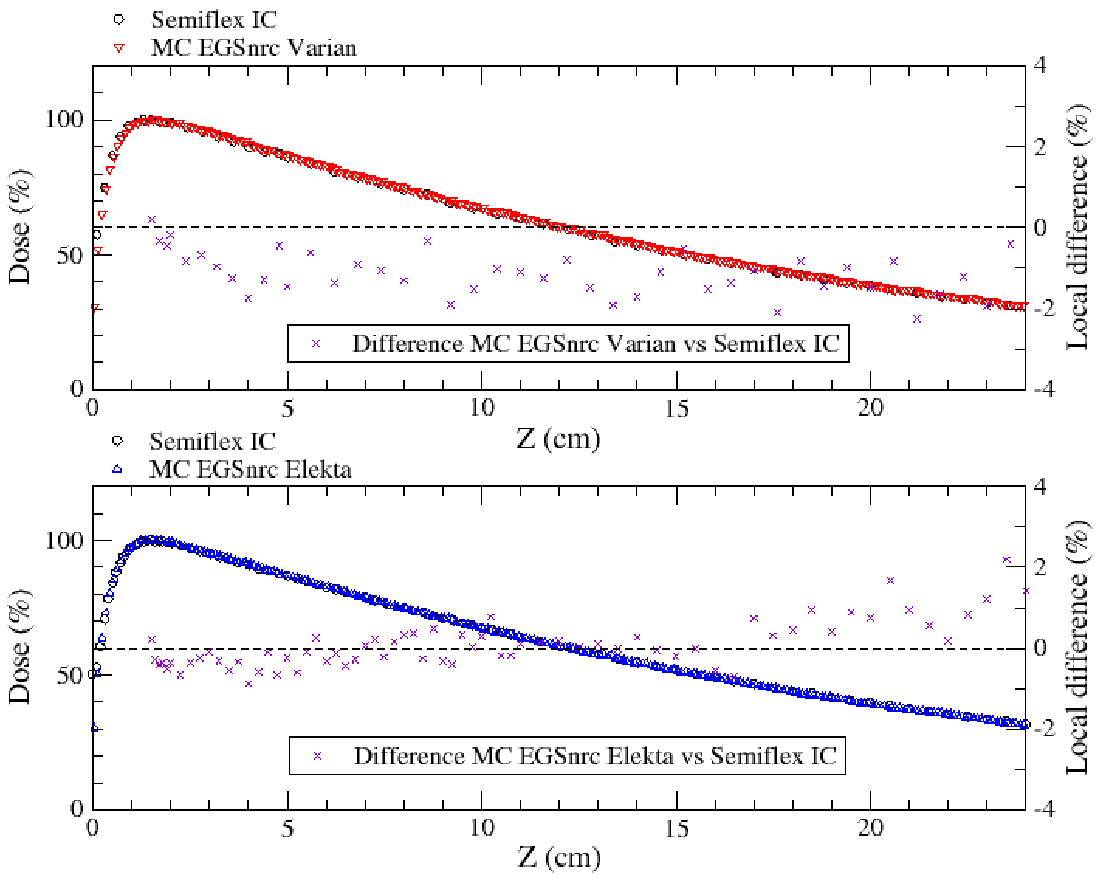

Figure S0. PDD measured with a semiflex IC and calculated with MCEGSnrc for the Varian21EX linac (upper plot) and Elekta Asses (lower plot). (x) represent the local differences relative to measurements (right axis). SSD=100 cm and SSD=95 cm setups for the Varian and Elekta linacs were used, respectively
