# Supplementary material for: Study of out‐of‐field dose in photon radiotherapy: A commercial treatment planning system versus measurements and Monte Carlo simulations
Source: Med Phys. 2020 Jul 16;47(9):4616–25. doi: 10.1002/mp.14356 (PMC7586840; doi:10.1002/mp.14356)
Supplement: Supplementary file 2 — Fig S1. Semiflex IC and MC in‐plane lateral profiles (left vertical axis) and local differences (right vertical axis) for the Varian linac. SSD=100 cm setup was used [Correction added on September 9, 2020, after first online publication: The Fig S1.caption have been corrected.] [file MP-47-4616-s002.pdf]

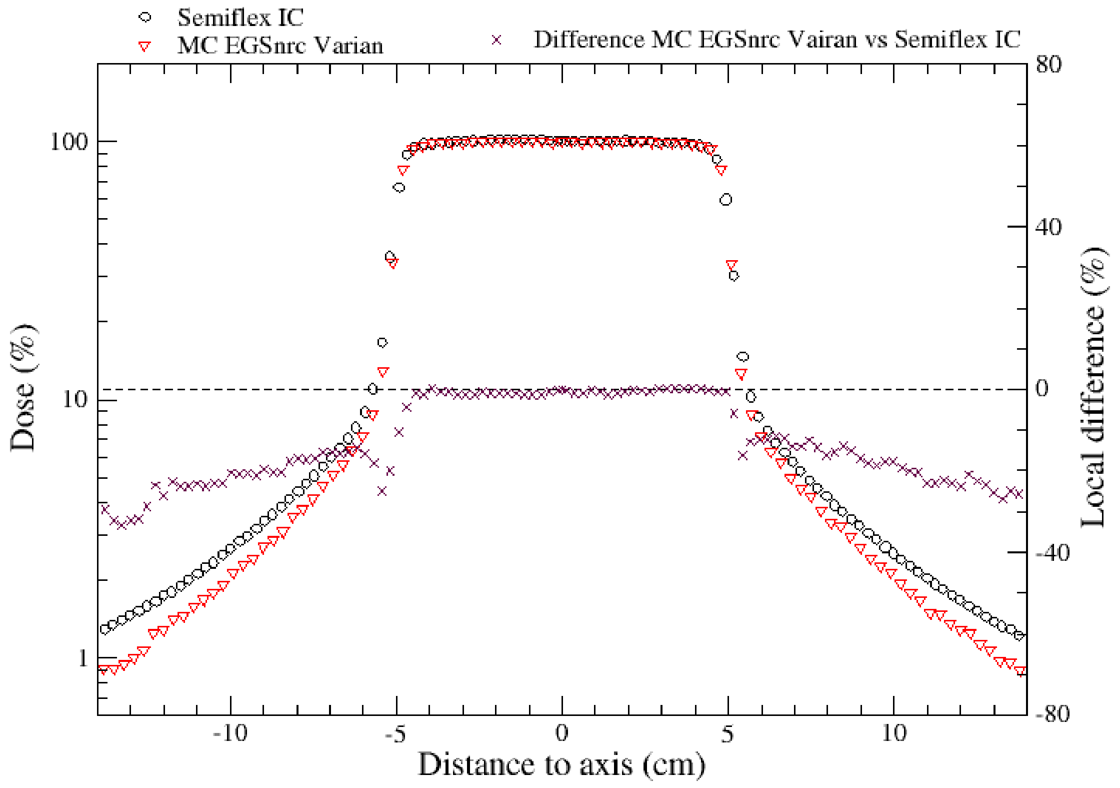

Figure S1. Semiflex IC and MC in-plane lateral profiles (left vertical axis) and local differences (right vertical axis) for the Varian linac. SSD=100 cm setup was used
