# Supplementary material for: Study of out‐of‐field dose in photon radiotherapy: A commercial treatment planning system versus measurements and Monte Carlo simulations
Source: Med Phys. 2020 Jul 16;47(9):4616–25. doi: 10.1002/mp.14356 (PMC7586840; doi:10.1002/mp.14356)
Supplement: Supplementary file 3 — Fig S2. Average energy of a 6 MV photon beam of a Varian 21EX linac. MC calculation was carried out at 5 cm depth in a water phantom (SSD = 100 cm). Solid lines correspond to the fitted interpolation model E=Ae(‐(x‐5)/t)+E0, where A, t, and E0 are the fitting parameters (A = 0.895 ± 0.037 MeV, t = 0.511 ± 0.076 cm−1, and E0 = 0.348 ± 0.016 MeV). [file MP-47-4616-s003.pdf]

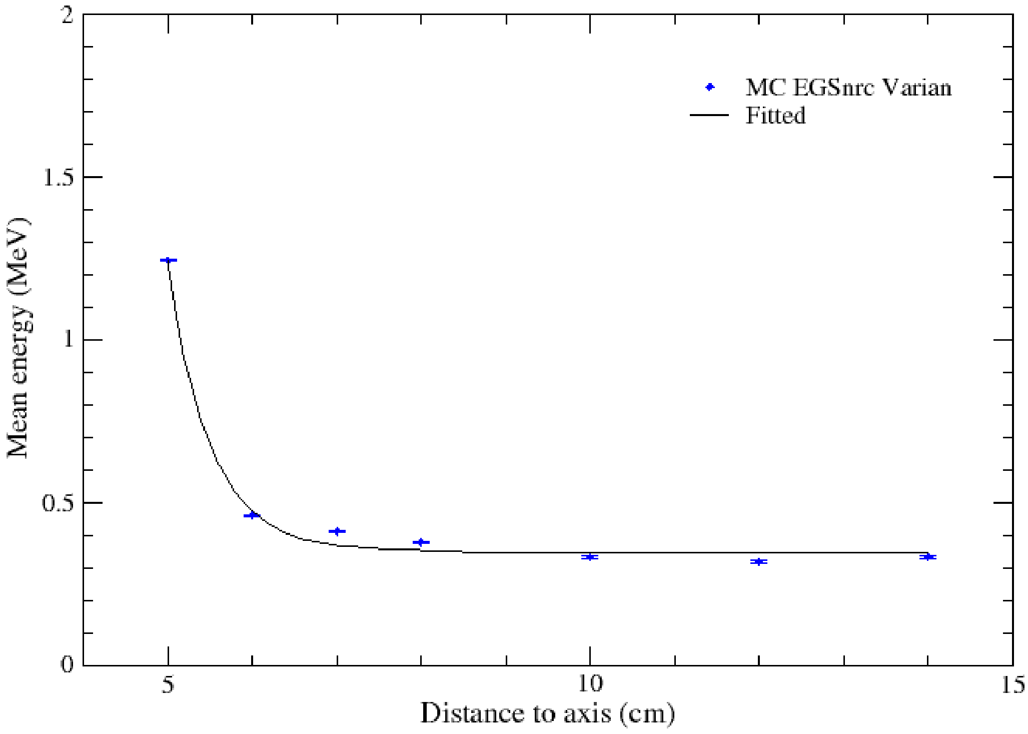

Figure S2. Average energy of a 6MV photon beam of a Varian 21EX linac. MC calculation were carried out at 5 cm depth in a water phantom (SSD=100 cm). Solid lines correspond to the fitted interpolation model $\langle E\rangle = A\,e^{\left(\frac{-(x-5)}{t}\right)} + E_0$, where A, t and E_0 are the fitting parameters (A = 0,895 ± 0,037 MeV, t = 0.511 ± 0,076 cm^{-1} and E_0 = 0.348 ± 0,016 MeV).
