# Supplementary material for: Study of out‐of‐field dose in photon radiotherapy: A commercial treatment planning system versus measurements and Monte Carlo simulations
Source: Med Phys. 2020 Jul 16;47(9):4616–25. doi: 10.1002/mp.14356 (PMC7586840; doi:10.1002/mp.14356)
Supplement: Supplementary file 4 — Fig S3. Half in‐plane dose profiles calculated with Eclipse and experimental data for the Varian linac. [file MP-47-4616-s004.pdf]

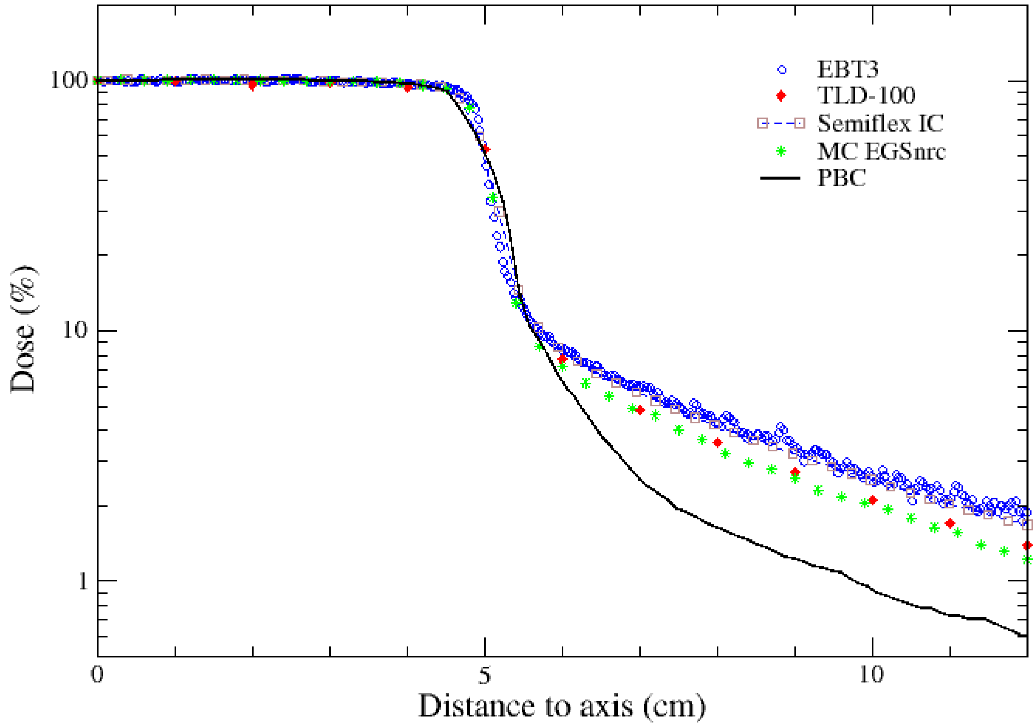

Figure S3. Half in-plane dose profiles calculated with Eclipse and experimental data for the Varian linac.
