# Supplementary material for: Study of out‐of‐field dose in photon radiotherapy: A commercial treatment planning system versus measurements and Monte Carlo simulations
Source: Med Phys. 2020 Jul 16;47(9):4616–25. doi: 10.1002/mp.14356 (PMC7586840; doi:10.1002/mp.14356)
Supplement: Supplementary file 5 — Fig S4. Profiles measured with EBT3 and TLDs under more similar conditions (i.e., EBT3 underneath the same bolus used for TLDs set up). [file MP-47-4616-s005.pdf]

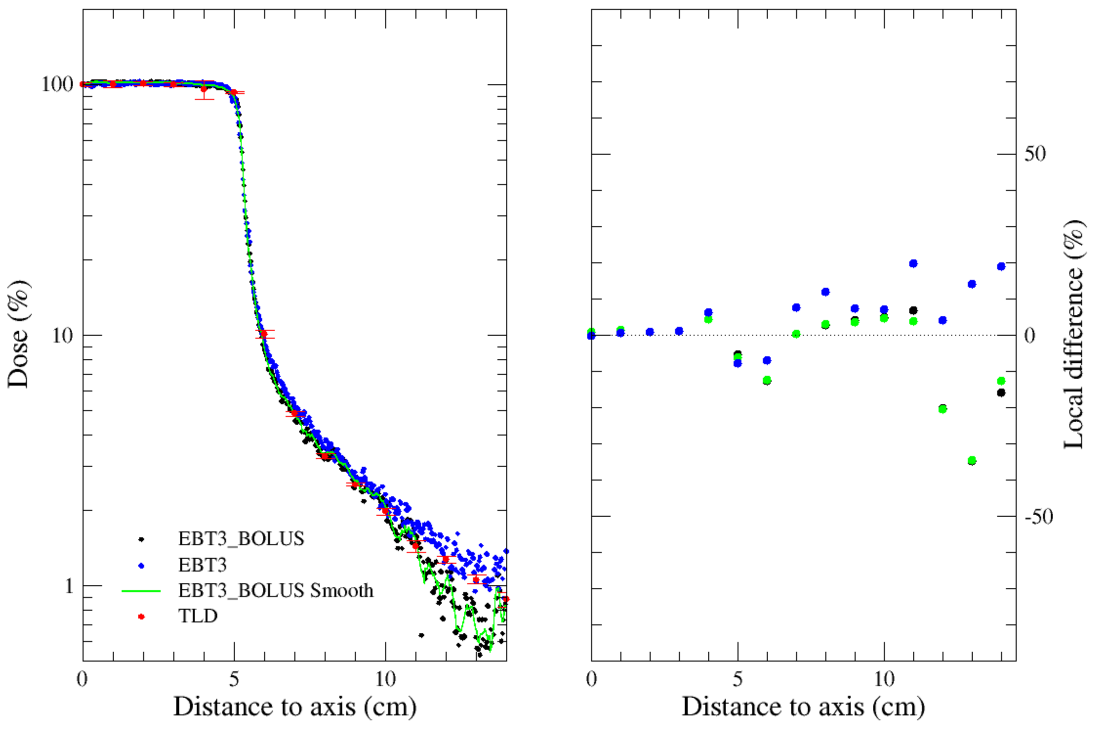

Figure S4. Profiles measured with EBT3 and TLDs under more similar conditions (i.e., EBT3 underneath the same bolus used for TLDs set up)
