# Supplementary material for: Study of out‐of‐field dose in photon radiotherapy: A commercial treatment planning system versus measurements and Monte Carlo simulations
Source: Med Phys. 2020 Jul 16;47(9):4616–25. doi: 10.1002/mp.14356 (PMC7586840; doi:10.1002/mp.14356)
Supplement: Supplementary file 6 — Table S1. Percentage difference in dose to organs with CCC. Table S2. Percentage difference in dose to organs with MCMonaco. [file MP-47-4616-s006.pdf]

Table S1: Percentage difference in dose to organs with CCC.

Organs	Mean dose (cGy)			Max dose (cGy)			Min Dose (cGy)		
	MC_{EGSnrc}	CCC	Difference (%)	MC_{EGSnrc}	CCC	Difference (%)	MC_{EGSnrc}	CCC	Difference (%)
Spinal cord	45,3	37,7	-17	196,1	177,3	-10	6,9	5,2	-25
Liver	46,7	24,5	-48	226,8	122,5	-46	10,3	5,3	-49
Stomach	11,7	8,4	-28	27,0	17,8	-34	7,1	4,0	-44
Pancreas	13,8	8,0	-42	24,8	13,6	-45	9,8	4,2	-57
Thyroid	16,9	11,1	-34	24,5	16,6	-32	11,2	8,2	-27
Small intestine	8,1	3,9	-52	30,4	16,6	-45	1,0	0,2	-84
Colon	10,8	5,7	-48	35,3	19,5	-45	1,0	0,1	-95
Brain	3,5	1,7	-51	7,1	11,1	57	1,2	0,1	-96
Bladder	0,9	0,3	-60	1,9	2,6	38	0,4	0,1	-86
Rectum	0,7	0,5	-26	1,0	5,1	432	0,5	0,2	-67
Prostate	0,3	0,2	-40	0,5	0,3	-44	0,2	0,2	0

Table S2: Percentage difference in dose to organs with MC_{Monaco}.

Organs	Mean dose (cGy)			Max dose (cGy)			Min Dose (cGy)		
	MC_{EGSnrc}	MC_{Monaco}	Difference (%)	MC_{EGSnrc}	MC_{Monaco}	Difference (%)	MC_{EGSnrc}	MC_{Monaco}	Difference (%)
Spinal cord	45,3	40,6	-10	196,1	241,3	23	6,9	0,9	-88
Liver	46,7	28,8	-38	226,8	150,0	-34	10,3	2,8	-73
Stomach	11,7	4,5	-61	27,0	14,9	-45	7,1	1,2	-84
Pancreas	13,8	5,2	-62	24,8	19,9	-20	9,8	2,6	-74
Thyroid	16,9	7,0	-59	24,5	11,8	-52	11,2	3,0	-74
Small intestine	8,1	1,6	-80	30,4	19,9	-35	1,0	0,1	-95
Colon	10,8	2,2	-79	35,3	29,6	-16	1,0	0,1	-95
Brain	3,5	0,5	-87	7,1	2,0	-72	1,2	0,1	-96
Bladder	0,9	0,1	-89	1,9	0,5	-76	0,4	0,1	-86
Rectum	0,7	0,1	-89	1,0	0,4	-63	0,5	0,1	-89
Prostate	0,3	0,1	-81	0,5	0,2	-67	0,2	0,1	-67
