# Supplementary material for: Study of out‐of‐field dose in photon radiotherapy: A commercial treatment planning system versus measurements and Monte Carlo simulations
Source: Med Phys. 2020 Jul 16;47(9):4616–25. doi: 10.1002/mp.14356 (PMC7586840; doi:10.1002/mp.14356)
Supplement: Supplementary file 7 — Annex S1. TLD procedure. [file MP-47-4616-s007.pdf]

Annex I

TLD procedure

The readout was carried out with a Thermo ScientificTM Harshaw TLD Model 3500 (Cleveland, Ohio) reader. Each TLD chip was characterized by an individual sensitivity correction factor (SCF). The standard deviation of the SCFs within the group was typically ±3%. Before irradiation, all crystals were annealed according to the following procedure: first, they were heated at 400°C for 1h, followed by a 2h cycle at 100°C. Then, they were fast cooled at room temperature. A pre-reading thermal treatment, consisting of a 10 min heating period at 100°C, followed by a 15 min fast cooling down was applied to the chips. The readout procedure consisted of a 100-300 °C temperature ramp at 10 °C/s. The calibration of the TLDs was done with a 6 MV photon beam and a 0.6 cm^3 Farmer chamber (PTW; Freiburg, Germany) calibrated at the University of Wisconsin-Radiation Calibration Laboratory.
